# Intradiaphragmatic extralobar pulmonary sequestration in adult

**DOI:** 10.1186/1749-8090-9-112

**Published:** 2014-06-20

**Authors:** Jang-Hoon Lee, Mi-Jin Kim

**Affiliations:** 1 Department of Thoracic and Cardiovascular Surgery, College of Medicine, Yeungnam University, Daemyeong 5-dong, Nam-gu, Daegu Zip code 705-717, Korea; 2 Department of Pathology, College of Medicine, Yeungnam University, Daegu, Korea

**Keywords:** Diaphragm, Pulmonary sequestration, Thoracoscopy

## Abstract

Extralobar pulmonary sequestrations may be located in intrathoracic or extrathoracic areas. Extrathoracic intradiaphragmatic extralobar pulmonary sequestrations are an extremely rare subset of bronchopulmonary sequestrations and there have been very few reported cases until now. We describe a 48-year-old Korean woman found to have left peridiaphragmatic lesion on computed tomography. We performed thoracoscopic surgery and successfully resected the tumor. Based on the histological findings, it was diagnosed as an intradiaphragmatic extralobar pulmonary sequestration. Postoperative course was uneventful. Intradiaphragmatic extralobar pulmonary sequestration in adult is extremely rare, so we report the case with a literature review.

## Background

Extralobar pulmonary sequestrations are most commonly found within the thoracic cavity, but have been described in extrathoracic areas as so called extrathoracic extralobar pulmonary sequestrations. Sequestrations within the diaphragm (intradiaphragmatic extralobar sequestration) are extremely rare. In this case, the tumor was resected using thoracoscopic surgery, and based on the histological findings, it was diagnosed as an intradiaphragmatic extralobar pulmonary sequestration. We herein report a case of an extrathoracic intradiaphragmatic extralobar pulmonary sequestration in a 48-year-old Korean woman.

## Case presentation

**Figure 1:**
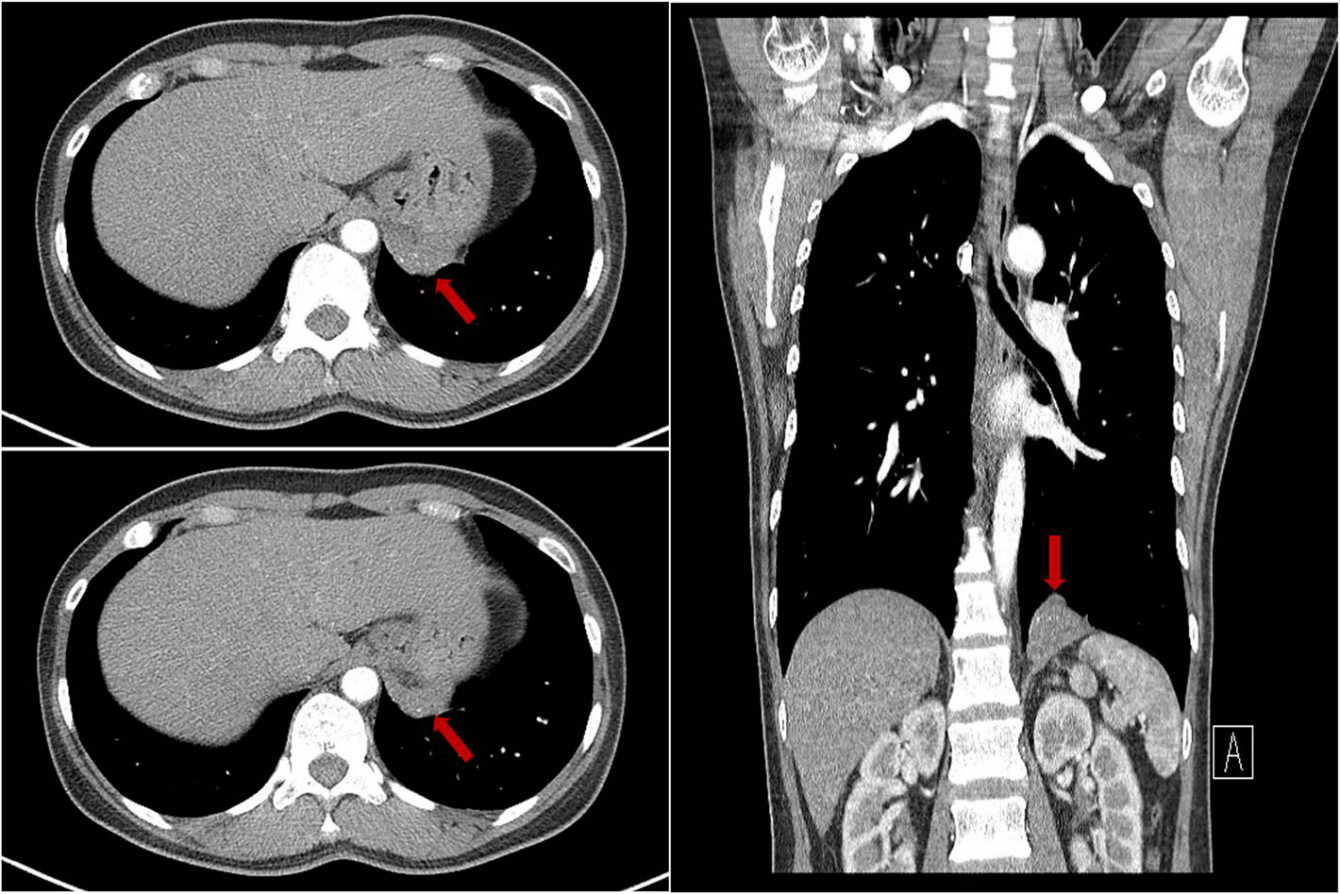
Preoperative chest computed tomography findings.

**Figure 2:**
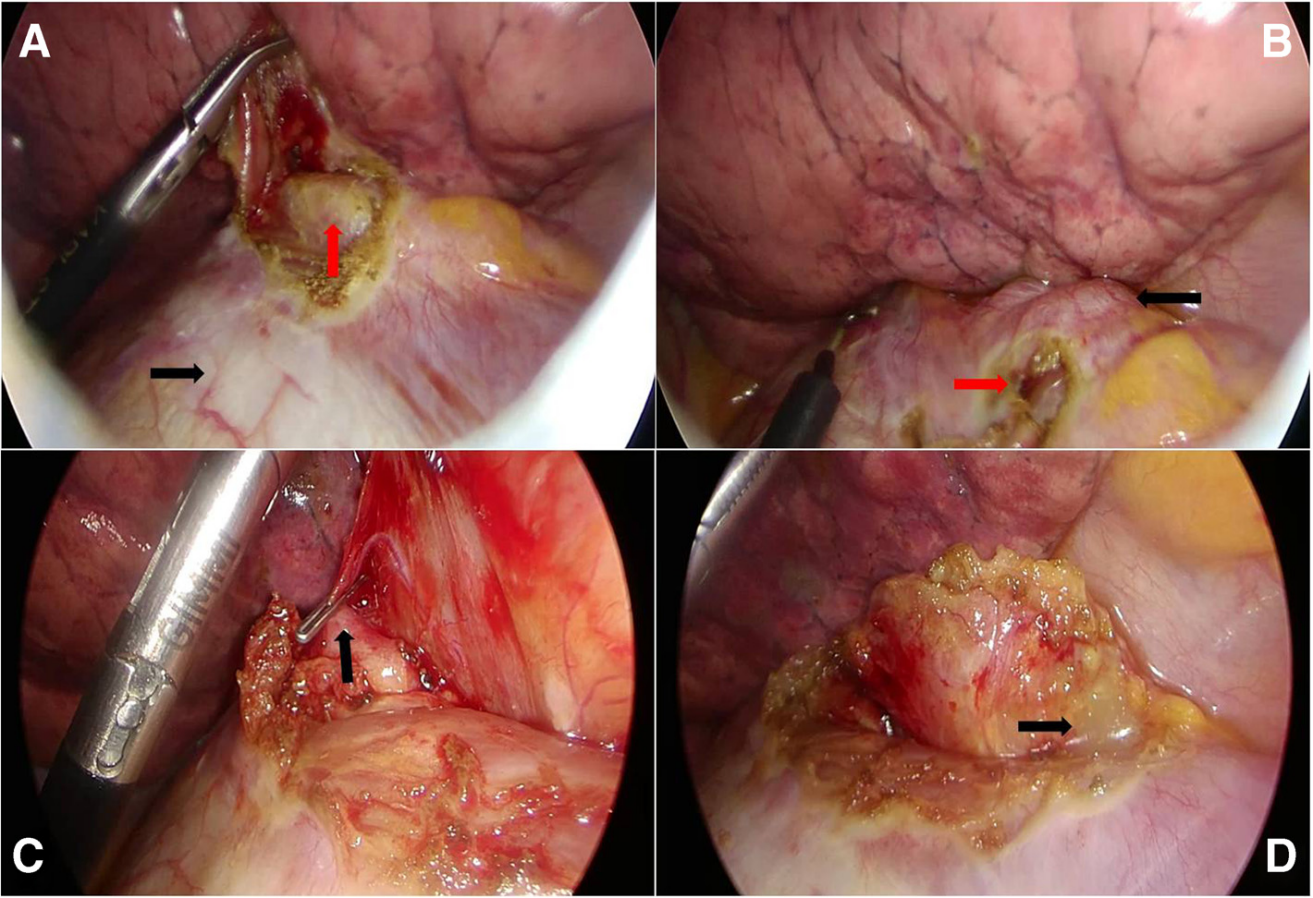
**Intraoperative thoracoscopic images. (A)** Incision of diaphragm (black arrow), Intradiaphragmatic mass (red arrow) was identified. **(B)** Diaphragmatic bulge (black arrow). Incision site of diaphragm (red arrow). **(C)** Small aberrant vessels were clipped (black arrow). **(D)** Yellowish mucoid materials were drained (black arrow).

**Figure 3:**
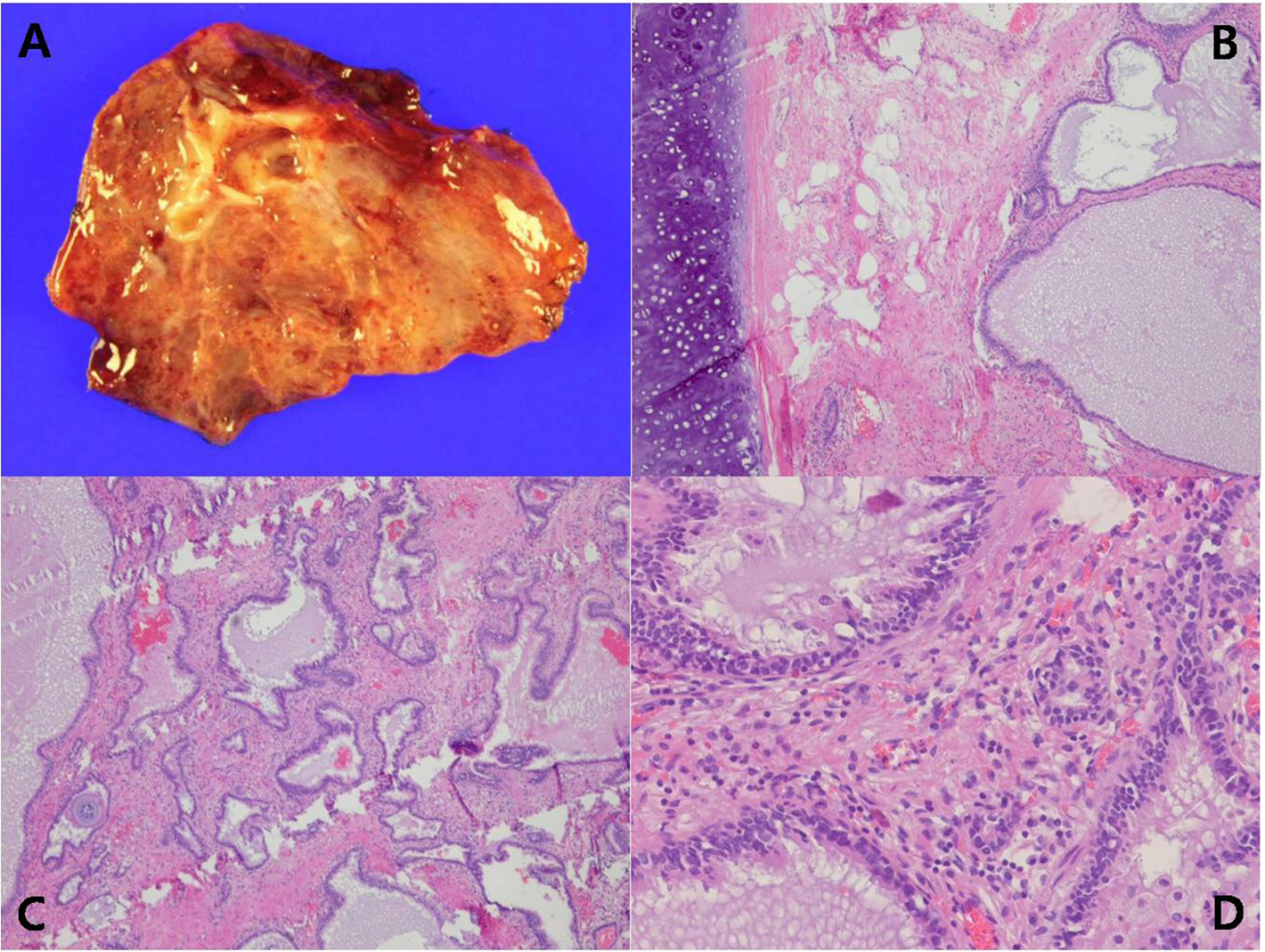
**Pathologic findings of the resected specimen. (A)** Gross findings. **(B)** Dilated mucin-filled airways and remnants of cartilaginous bronchi (x 100, hematoxylin and eosin stain). **(C)** Normal lung tissue is not observed (x 100, hematoxylin and eosin stain). **(D)** Dilated airways are lined by bronchiolar type epithelium (x 200, hematoxylin and eosin stain).

A 48-year-old Korean female patient presented with an abnormal mass lesion that was detected by abdominal computed tomography in a visit to our hospital. She had experienced intermittent abdominal pain for several months. She had no other specific past medical history and no history of trauma. The patient’s vital signs were stable and laboratory tests were normal. Chest X-ray showed no abnormal findings and the computed tomography of her chest showed a 4-cm-sized round mass with areas of calcification in the left hemidiaphragmatic area (Figure 1). After a review of the diagnostic imaging, we were still unable to localize the mass, but we concluded that the lesion was most likely located in the left pleural space based on its proximity to the diaphragm. We decided to remove the mass. The patient was taken to the operating room for thoracoscopic surgery. General anesthesia with double lumen endotracheal tube intubation and one lung ventilation was done. Two 5-mm ports and one 10-mm port were placed in the left chest (fifth intercostal space in the midclavicular line, sixth intercostal space in the anterior axillary line, and eighth intercostal space in the posterior axillary line) for the thoracoscopic approach. No mass was visualized in the pleural space, but a bulge was visualized in the diaphragm consistent with the location of the lesion noted on chest computed tomography. The diaphragm was opened with electrocautery around the mass lesion. Then we identified the mass in the diaphragm (Figure 2). The mass was adhered to the crucial fibers of the diaphragm but was relatively well marginated. We dissected carefully, and a small feeding vessel was noted and clipped. The dissection was relatively easy and the mass was removed. The diaphragm defect was closed with interrupted polyester sutures and one chest tube was placed. The postoperative course was uneventful. The chest tube was removed on the third postoperative day and the patient was discharged the following day. The specimen measured about 4 cm in diameter, 9.4 gram in weight, and was well-defined and reddish. Cut sections of the mass showed sponge-like appearance with cartilage and yellow-colored mucoid materials. Histologic evaluation of the specimen was consistent with the diagnosis of an extralobar sequestration (Figure 3).

### Discussion

Pulmonary sequestration was first defined by Pryce in 1949 [1] as characterized by a non-functional lung without communication with the bronchial tree and the presence of an aberrant blood supply. On the basis of morphological patterns, they are divided into two types: intralobar and extralobar. An intralobar sequestration shares the same pleura with normal lung, but an extralobar sequestration has a separate pleura. Intralobar sequestrations are more common (75-85% of cases), while only 25% are extralobar sequestrations [2,3]. Extralobar sequestrations are most commonly found in the thorax, usually on the left side [4]. Only 10-15% of extralobar sequestrations are located in the abdomen [3,5]. Usually, extrathoracic extralobar pulmonary sequestrations are infradiaphragmatic, masquerading as suprarenal masses [4,6-8]. Intradiaphragmatic extralobar pulmonary sequestration is rare and there have been very few reported cases until now [9-11]. The location of extralobar pulmonary sequestrations in the diaphragm sheds light on the relationship between the embryology of sequestration, diaphragm, and lung. The pleuroperitoneal folds form and coalesce the primordial diaphragm from the body wall during the 9th to 12th weeks of gestation; therefore, a bronchopulmonary sequestration that arises during this period may have a higher chance of forming within the diaphragm [10]. True intradiaphragmatic pulmonary sequestrations are rare and all reported cases have been younger than two year old. In the present case, the patient was a 48-year-old female. This is the first case found in an adult. Pulmonary sequestration can usually be identified by diagnostic imaging as a soft tissue mass with an aberrant blood supply [9]. In our case, computed tomography of the patient showed a soft tissue mass but did not reveal an aberrant blood supply. The imaging diagnosis of intradiaphragmatic pulmonary sequestration is not easy. In 2009, Meier et al. [11] described the “split hemidiaphragm sign” as a radiologic finding of two leaflets of diaphragmatic muscle surrounding a soft tissue mass on computed tomography. This is helpful for preoperative diagnosis of this rare disease. However, in our case, we could not identify such findings on our patient’s computed tomographic scan. The appropriate management of extrathoracic extralobar pulmonary sequestration remains controversial. Some authors advocate expectant management without resection [12,13]. Other authors recommend embolization of the systemic artery as a treatment option [14]. However, most authors recommend surgical removal, especially for extrathoracic lesions, due to concern for infection, malignant degeneration, and difficult differentiation from another neoplasm [15,16]. In this report we could not diagnose the extrathoracic pulmonary sequestration. We chose surgical removal to allow differentiation from another neoplasm such as teratoma or certain types of malignancy. For surgical removal of intradiaphragmatic extralobar pulmonary sequestration, thoracoscopy is recommended. McAteer et al. [9] described that thoracoscopy provides excellent visualization of intradiaphragmatic masses and easy access for surgical resection. They also noted that the thoracoscopic approach allows careful dissection of the mass away from the diaphragm and primary repair of the resulting defect. In our case, we performed the operation via thoracoscopy and dissection of the mass from diaphragm and primary repair of the defect were not difficult.

## Conclusions

A case of intradiaphragmatic extralobar pulmonary sequestration is rare. The patients previously reported with such lesions were neonates or younger than two year old. To our knowledge, this is the only case of intradiaphragmatic extralobar pulmonary sequestration in an adult. We report the intradiaphragmatic extralobar pulmonary sequestration and its successful removal by thoracoscopic surgery along with literature reviews.

## Consent

Written informed consent was obtained from the patient for publication of this case report and any accompanying images. A copy of the written consent is available for review by the Editor-in-Chief of this journal.

## Competing interests

The authors declare that they have no competing interests.

## Acknowledgements

We gently appreciate the staffs of Radiology, YeungNam University Medical Center.

## Authors’ contributions

JH and MJ wrote the draft of the manuscript and obtained the written consent. JH performed the literature review and participated in the manuscript writing and helped to the final writing of the paper and gave final approval of the manuscript. All authors have read and approved the final manuscript.
